# Visual Attention to Economic Information in Simulated Ophthalmic Deficits: A Remote Eye-Tracking Study

**DOI:** 10.3390/jemr18050050

**Published:** 2025-10-02

**Authors:** Cansu Yuksel Elgin, Ceyhun Elgin

**Affiliations:** 1 Department of Ophthalmology, Istanbul University-Cerrahpasa, Istanbul 34098, Türkiye; 2 Department of Economics, Bogazici University, Istanbul 34342, Türkiye; ceyhun.elgin@bogazici.edu.tr

**Keywords:** visual field deficits, eye tracking, economic decision-making, visual attention, cognitive load, webcam-based eye tracking, information processing, visual impairment, financial accessibility

## Abstract

This study investigated how simulated ophthalmic visual field deficits affect visual attention and economic information processing. Using webcam-based eye tracking, 227 participants with normal vision recruited through Amazon Mechanical Turk were assigned to control, central vision loss, peripheral vision loss, or scattered vision loss simulation conditions. Participants viewed economic stimuli of varying complexity while eye movements, cognitive load, and comprehension were measured. All deficit conditions showed altered oculomotor behaviors. Central vision loss produced the most severe impairments: 43.6% increased fixation durations, 68% longer scanpaths, and comprehension accuracy of 61.2% versus 87.3% for controls. Visual deficits interacted with information complexity, showing accelerated impairment for complex stimuli. Mediation analysis revealed 47% of comprehension deficits were mediated through altered attention patterns. Cognitive load was significantly elevated, with central vision loss participants reporting 84% higher mental demand than controls. These findings demonstrate that visual field deficits fundamentally alter economic information processing through both direct perceptual limitations and compensatory attention strategies. Results demonstrate the feasibility of webcam-based eye tracking for studying simulated visual deficits and suggest that different types of simulated visual deficits may require distinct information presentation strategies.

## 1. Introduction

The intersection of ophthalmology and economics represents an emerging frontier in understanding how visual impairments affect financial decision-making and economic well-being. Visual field deficits, such as those caused by glaucoma, macular degeneration, and diabetic retinopathy, affect millions of individuals worldwide and can profoundly impact their ability to process and interpret visual information necessary for economic activities. While extensive research has documented the clinical manifestations and progression of these conditions, surprisingly little attention has been paid to their specific effects on economic information processing and decision-making capabilities.

Recent advances in eye-tracking technology have opened new avenues for investigating the relationship between visual attention and cognitive processing. Eye movements serve as a window into cognitive processes, revealing not only where individuals look but also how they allocate their attention and process information. In the context of economic decision-making, visual attention patterns have been shown to predict choice behavior and reveal underlying decision strategies. A study [1] has demonstrated that fixation patterns during economic choice tasks directly influence value computation and subsequent decisions, with longer fixations on an option increasing the probability of choosing that option. This fundamental link between visual attention and economic decision-making suggests that disruptions to normal visual processing, such as those caused by ophthalmic conditions, could have significant implications for economic behavior.

The advent of remote eye-tracking technologies, particularly webcam-based systems deployable through crowdsourcing platforms like Amazon Mechanical Turk (MTurk), has revolutionized the scalability and accessibility of eye-tracking research. These technologies enable researchers to collect eye movement data from diverse populations without the geographical and logistical constraints of traditional laboratory-based studies. The validity of webcam-based eye tracking for research purposes has been established in recent studies, with one [2] demonstrating that online eye-tracking experiments can achieve data quality comparable to laboratory-based systems for many experimental paradigms. This methodological advancement makes it feasible to study visual attention patterns in large, diverse samples, including populations with various ophthalmic conditions who might face barriers to participating in traditional laboratory studies.

The theoretical foundation for understanding how visual impairments affect economic information processing draws from three converging frameworks. Cognitive load theory [3] predicts that visual impairments increase extraneous cognitive load, leaving fewer resources for germane processing of economic information. This theory has been extensively validated in educational contexts [4] and provides a framework for understanding why visual deficits may disproportionately affect complex information processing. Vision science research on oculomotor adaptation demonstrates that individuals with visual field deficits develop compensatory viewing strategies, including preferred retinal locus development [5] and systematic scanning patterns [6], though these adaptations may be suboptimal for certain information types. From behavioral economics, models of bounded rationality [7,8] suggest that processing constraints systematically affect decision quality, with implications for how visual limitations might bias economic choices.

The economic implications of visual impairments extend far beyond the direct costs of medical care. Individuals with visual field deficits may experience challenges in interpreting financial documents, analyzing investment information, comparing prices, and making informed consumer choices. These difficulties can lead to suboptimal financial decisions, reduced economic participation, and diminished quality of life. Understanding how specific types of visual impairments affect the processing of economic information is crucial for developing appropriate accommodations and support systems. Moreover, as financial services increasingly move online and rely on visual interfaces, the ability to effectively process visual economic information becomes even more critical for full economic participation.

The current study addresses a significant gap in the literature by examining how simulated ophthalmic visual field deficits affect visual attention patterns when economic information is viewed. By using digital simulation techniques, we can systematically investigate the effects of different types of visual impairments while controlling for confounding factors that might arise in studies with clinical populations. This approach allows us to isolate the specific effects of visual field deficits on attention and information processing, providing insights that can inform both clinical practice and the design of economic information systems.

This study addresses three primary research questions with corresponding testable hypotheses:

Research Question 1: How do different types of simulated visual field deficits (central, peripheral, scattered) affect eye movement patterns when economic information is viewed?

**Hypothesis** **1a.** *Participants with simulated central vision loss will exhibit longer fixation durations and larger saccade amplitudes compared to controls, reflecting compensatory peripheral viewing strategies.*

**Hypothesis** **1b.** *Participants with simulated peripheral vision loss will show shorter saccade amplitudes and more concentrated fixation patterns within the central visual field.*

**Hypothesis** **1c.** *Participants with scattered vision loss will demonstrate more erratic scanning patterns with increased refixations and longer scanpaths.*

Research Question 2: Do visual field deficits interact with information complexity to affect comprehension of economic information?

**Hypothesis** **2.** *The negative impact of visual field deficits on comprehension accuracy will be magnified for complex economic stimuli, demonstrating a multiplicative rather than additive effect.*

Research Question 3: What is the relationship between altered visual attention patterns and subjective cognitive load in individuals with simulated visual field deficits?

**Hypothesis** **3a.** *Participants with visual field deficits will report significantly higher cognitive load compared to controls.*

**Hypothesis** **3b.** *Objective measures of visual attention (fixation duration, scanpath efficiency) will mediate the relationship between visual condition and comprehension performance. These hypotheses are grounded in cognitive load theory, which predicts that visual processing constraints will consume cognitive resources otherwise available for information comprehension and decision-making.*

Our research builds upon established frameworks in both vision science and behavioral economics. From the vision science perspective, we draw on models of visual attention and eye movement control that explain how individuals with visual field deficits adapt their viewing strategies to compensate for their impairments. From the behavioral economics perspective, we incorporate theories of bounded rationality and cognitive load that predict how information processing constraints affect decision-making. In a somewhat recent study [9], an integrated framework was proposed showing how visual salience and cognitive factors jointly determine attention allocation during complex decision tasks, highlighting the importance of considering both perceptual and cognitive factors in understanding economic information processing.

The potential applications of this research extend to multiple domains, including financial literacy education, the design of accessible financial interfaces, and policy recommendations for protecting vulnerable populations in economic contexts. By identifying specific challenges faced by individuals with visual impairments when processing economic information, we can work toward creating more inclusive economic systems that accommodate diverse visual capabilities and ensure equitable access to financial information and services.

## 2. Related Literature

The study of visual attention in economic decision-making has evolved significantly over the past two decades, revealing intricate relationships between eye movements, cognitive processing, and choice behavior. This literature review examines three interconnected domains relevant to understanding how ophthalmic deficits affect economic information processing: visual field deficits and eye movement adaptations, visual attention in economic decision-making, and the emergence of remote eye-tracking methodologies. Visual field deficits fundamentally alter how individuals explore and process visual information. Patients with conditions such as glaucoma, age-related macular degeneration, and hemianopia develop compensatory eye movement strategies to overcome their visual limitations. A pioneering study [10] examined eye movements in glaucoma patients during natural viewing tasks, finding that patients with bilateral visual field defects exhibited significantly different scanning patterns compared to healthy controls, including increased fixation durations and reduced saccade amplitudes. These adaptations, while potentially beneficial for general visual exploration, may have unintended consequences when processing information-dense materials such as financial documents.

The relationship between visual field loss and reading behavior has been extensively studied, with important implications for processing textual economic information. A study [11] investigated reading performance in patients with central field loss due to macular degeneration, demonstrating that these individuals developed eccentric viewing strategies but experienced significantly reduced reading speeds and increased cognitive load. This finding is particularly relevant for economic contexts where rapid processing of numerical and textual information is often required for effective decision-making.

Recent neuroimaging studies have examined cortical reorganization following visual field loss. Baseler et al. [12] used functional magnetic resonance imaging to demonstrate that large-scale remapping of the visual cortex is absent in adult humans with macular degeneration, finding no evidence of cortical remapping following retinal lesions acquired in adulthood, suggesting that compensatory strategies rely primarily on altered attention allocation rather than fundamental cortical reorganization. How these attention-based adaptations influence higher-level cognitive processes involved in economic decision-making remains largely unexplored.

The role of visual attention in economic choice has emerged as a central topic in neuroeconomics and behavioral economics. Eye-tracking studies have revealed that attention is not merely a passive reflection of preference but actively shapes the decision-making process. Orquin and Mueller Loose conducted a comprehensive meta-analysis of eye-tracking studies in decision-making, finding consistent evidence that attention, as measured by fixation duration and frequency, predicted choice outcomes across various decision contexts [13]. Their analysis highlighted the importance of understanding attention allocation patterns for predicting and potentially influencing economic behavior.

The complexity of financial information presents unique challenges for visual processing and decision-making. Rubaltelli et al. examined how individuals process investment fund information, finding that visual complexity significantly affected both attention patterns and investment choices [14]. Participants spent more time fixating on simpler graphical representations compared to complex tables, and these attention patterns correlated with their final investment decisions. This research suggests that the format and visual complexity of economic information can profoundly influence how it is processed and acted upon.

The concept of cognitive load is particularly relevant when considering how visual impairments might affect economic decision-making. A study [15] investigated the effects of cognitive load on financial decision-making, demonstrating that increased cognitive demands led to more reliance on heuristics and potentially suboptimal choices. For individuals with visual field deficits who must exert additional effort to extract visual information, this cognitive load effect may be amplified, potentially leading to systematic biases in economic decisions.

The development of webcam-based eye-tracking technology has democratized access to eye movement research, enabling studies with larger and more diverse populations. Papoutsaki et al. developed WebGazer, an open-source eye-tracking library that operates entirely within web browsers, achieving accuracy levels suitable for many research applications [16]. Their validation studies demonstrated that while webcam-based tracking cannot match the precision of dedicated eye-tracking hardware, it provides sufficient accuracy for studying gross attention patterns and fixation behaviors relevant to economic decision-making tasks.

The integration of eye-tracking capabilities with online crowdsourcing platforms has opened new methodological possibilities. Xu et al. conducted one of the first large-scale webcam-based eye-tracking studies using Amazon Mechanical Turk, successfully collecting data from over 1000 participants across diverse demographic groups [17]. Their work established important precedents for data quality control and validation in remote eye-tracking studies, providing a methodological foundation for subsequent research in this area.

Despite the growing bodies of literature on visual field deficits, eye movements in economic decision-making, and remote eye-tracking methodologies, there remains a significant gap in understanding how ophthalmic conditions specifically affect the processing of economic information. Previous studies have examined these domains largely in isolation, without considering their intersection. The current study addresses this gap by combining insights from vision science, behavioral economics, and modern eye-tracking technology to investigate how simulated visual field deficits influence attention to and comprehension of economic information. This integration is essential for developing evidence-based recommendations for making financial information more accessible to individuals with visual impairments.

Research on cognitive load and visual processing has established that visual impairments create dual-task conditions where individuals must simultaneously manage compensatory viewing strategies while processing information content [18]. Studies using dual-task paradigms have shown that cognitive resources allocated to visual compensation reduce performance on concurrent cognitive tasks [19], providing a theoretical basis for our expectation that visual deficits will impair economic information processing. The intersection of visual impairment and financial decision-making represents an emerging research area. Bateman et al. [20] demonstrated that visual presentation format significantly affects investment decisions, while Kozup et al. [21] showed that information complexity interacts with processing constraints to influence choice quality. Research on numeracy and financial decision-making [22,23] suggests that difficulties extracting numerical information, as might be expected with visual impairments, can lead to systematic decision biases. Accessibility research has identified specific challenges faced by individuals with visual impairments when interacting with financial services. Shinohara and Wobbrock [24] documented that current accessibility standards are often inadequate for complex interactive tasks, while studies of Section 508 compliance have revealed gaps between technical accessibility and functional usability [25]. The literature suggests that our investigation of economic information processing addresses a significant practical need.

## 3. Materials and Methods

This study employed a between-subject experimental design to investigate how simulated ophthalmic visual field deficits affect visual attention patterns and cognitive processing when economic information is viewed. The research protocol received approval from the American University in Bulgaria (AUBG) Human Subject Review Board (No: ELG072104) prior to implementation in 2024, ensuring compliance with ethical standards for human subject research. We recruited 240 participants through Amazon Mechanical Turk (MTurk), with 60 participants randomly assigned to each of four conditions: control (no visual impairment), central vision loss simulation, peripheral vision loss simulation, and scattered vision loss simulation. Participants were required to be between 18 and 65 years of age, have normal or corrected-to-normal vision, possess a webcam-enabled computer with a stable internet connection, and be fluent in English. We excluded individuals with diagnosed ophthalmic conditions, those with neurological disorders affecting vision, or those using medications known to affect eye movements. To ensure data quality, we restricted participation to MTurk workers with approval ratings above 95% and at least 1000 completed tasks.

The sample size was determined through a power analysis based on effect sizes reported in previous eye-tracking studies of visual attention in decision-making contexts. Given the moderate to large effect sizes typically observed in such studies and accounting for potential data loss due to poor tracking quality, we aimed for 60 participants per condition to achieve 80% power to detect medium effect sizes (d = 0.5) at α = 0.05.

The study utilized a 4 (visual condition: control, central loss, peripheral loss, scattered loss) × 3 (information complexity: low, medium, high) mixed design, with visual condition as a between-subject factor and information complexity as a within-subject factor. This design allowed us to examine both the main effects of visual impairment type and the interaction between visual deficits and information complexity on attention patterns and comprehension.

Economic visual stimuli consisted of investment performance charts, financial comparison tables, risk–return scatter plots, and portfolio allocation pie charts. Each stimulus category was presented at three complexity levels. Low-complexity stimuli contained 3–5 data points with simple labels, medium-complexity stimuli included 8–12 data points with additional annotations, and high-complexity stimuli featured 15–20 data points with multiple variables and detailed legends. All stimuli were created using standardized templates to ensure consistency in visual design while varying informational content.

Visual field deficit simulations were implemented using real-time image processing algorithms applied through JavaScript in the participant’s web browser. The central vision loss simulation created a semi-transparent gray circular scotoma with a 10-degree radius covering central vision, mimicking advanced macular degeneration. The peripheral vision loss simulation restricted the visible field to the central 40 degrees, simulating severe glaucomatous field loss. The scattered vision loss simulation applied multiple small scotomas distributed across the visual field, representing patchy vision loss patterns. These simulations were validated against clinical descriptions and adjusted based on pilot testing to ensure they adequately represented the intended visual deficits while remaining viewable for experimental purposes.

Participants accessed the study through a secure web interface after accepting the task on MTurk. The experimental session began with detailed instructions and informed consent procedures. Participants then completed a demographic questionnaire and a brief visual acuity check using a web-based Snellen chart equivalent to ensure adequate vision for the task.

The webcam calibration procedure followed protocols established by [16] for WebGazer-based eye tracking. Participants completed a 9-point calibration sequence, fixating on targets presented sequentially at different screen locations. The calibration was repeated if the estimated accuracy exceeded 2° visual angle or 5% of screen width. Drift checks occurred every 6 trials, with recalibration required if accuracy degraded beyond 3° (success rate: 89%). Failed calibrations resulted in participant exclusion (*n* = 13 total: Control = 3, Central = 4, Peripheral = 3, Scattered = 3), yielding final sample sizes of *n* = 57, 56, 57, and 57, respectively. To maintain tracking accuracy throughout the session, we implemented periodic drift checks between blocks of trials. No video frames or facial images were stored. Only anonymized gaze coordinates were retained locally on encrypted servers for 24 months with restricted access limited to research personnel. Participants provided informed consent for data collection and temporary retention procedures.

Following successful calibration, participants were randomly assigned to one of the four visual conditions. The assigned visual field simulation was then applied to all subsequent stimuli. Participants completed 36 experimental trials (12 stimuli × 3 complexity levels) presented in randomized order. Each trial began with a central fixation cross displayed for 1000 ms, followed by the economic stimulus presented for 30 s. Participants were instructed to study the information carefully as they would be asked questions about it. After each stimulus, participants answered three comprehension questions and rated their subjective cognitive load using the NASA Task Load Index [26]. NASA-TLX responses were collected on 0–100 scales using raw scoring without weighting procedures. We used unweighted averaging of the six subscales to compute total cognitive load scores, following Hart and Staveland’s original recommendation that raw TLX scoring provides adequate sensitivity while avoiding the complexity and time requirements of the weighting procedure. This approach has been validated in numerous studies and is appropriate for between-group comparisons where relative differences in cognitive load are the primary interest.

Eye-tracking metrics were computed using custom JavaScript algorithms processing the raw gaze coordinates provided by the WebGazer API. Raw coordinates (30 Hz sampling) were smoothed using a 3-point moving average filter. Fixations were defined using dispersion-based criteria: gaze points remaining within 1° visual angle for a minimum 100 ms duration. No additional resampling or denoising was applied beyond the 3-point moving average filter for coordinate smoothing. Saccade amplitude and scanpath length (cumulative inter-fixation distances) were normalized to screen dimensions. Areas of Interest (AOIs) were defined as hierarchical regions: titles (top 15%), axis labels (left 10%, bottom 10%), legends (right 15%), and data regions (remaining central area). AOI boundaries were defined hierarchically to handle potential overlaps across different screen configurations. When fixations fell within overlapping regions, assignment followed the following precedence order: (1) titles (highest priority), (2) legends, (3) axis labels, (4) data regions (lowest priority). This hierarchy ensured that fixations on critical text elements were not misclassified as data region viewing. Boundary detection used pixel-precise hit testing, with fixations assigned to the highest-priority AOI containing the gaze coordinates. We computed dwell time percentage and transition probabilities between AOIs to assess information extraction strategies.

Survey measures included the NASA-TLX for cognitive load assessment across six dimensions: mental demand, physical demand, temporal demand, performance, effort, and frustration. Economic comprehension was assessed through multiple-choice questions targeting factual recall, trend identification, and comparative judgments relevant to each stimulus. Response accuracy and confidence ratings were recorded for each question.

Data preprocessing involved removing trials with excessive data loss (>30% missing gaze data) and participants with poor overall tracking quality. Statistical analyses were conducted in R (version 4.3.2). Linear mixed-effects models were fitted at the trial level using lme4 (version 1.1-35) [27] with restricted maximum likelihood (REML) estimation. Model selection began with maximal random-effects structures following Barr et al.’s recommendations: Outcome ~ Condition × Complexity + (1 + Complexity|Participant), followed by simplification based on convergence warnings and singular fit detection. Final models retained random intercepts for all participants and random slopes for complexity where supported by likelihood ratio tests (*p* < 0.05) and where models converged successfully. Comprehension accuracy, being binary at the trial level (correct/incorrect), was analyzed using generalized linear mixed-effects models with a binomial distribution and logit link function. Primary analyses used glmer() from lme4 with the following specification: Accuracy ~ Condition × Complexity + (1|Participant). We report odds ratios (ORs) with 95% confidence intervals as the primary effect size measure. For ease of interpretation and comparison with previous literature, we also conducted secondary analyses using linear probability models (LPMs) on the same trial-level data. Model fit was assessed using marginal R^2^ (variance explained by fixed effects) and conditional R^2^ (variance explained by fixed and random effects) calculated via the MuMIn package, along with intraclass correlation coefficients (ICCs) computed as σ^2^ participant/(σ^2^ participant + π^2^/3) for logistic models. For continuous eye-tracking outcomes, we used lmer() with the following model specification: Outcome ~ Condition × Complexity + (1|Participant), with random slopes for complexity included only when supported by the data. For count outcomes (e.g., fixation frequency), we used glmer() with Poisson family and log link, with overdispersion assessed through comparison of residual deviance to degrees of freedom. For hypothesis testing, we used likelihood ratio tests (implemented via anova() function) comparing nested models with and without the effect of interest, reporting χ^2^ statistics with associated degrees of freedom. Individual contrasts and post hoc comparisons were computed using emmeans (version 1.8.9) with Kenward–Roger degrees of freedom approximation via pbkrtest (version 0.5-0.2), reporting *t*-statistics with software-computed degrees of freedom. Collinearity among fixed effect predictors was assessed using variance inflation factors, with all VIFs remaining below 2.5, indicating acceptable levels of multicollinearity. For count outcomes (fixation frequency), we fitted Poisson models using glmer() and assessed overdispersion by comparing residual deviance to degrees of freedom. When overdispersion was detected (deviance/df > 1.5), we refitted using negative binomial models via glmer.nb(). For continuous outcomes with positive skew (fixation duration, scanpath length), we examined log-transformed models alongside untransformed analyses. Model selection favored log-transformed fixation duration (AIC difference = 47.3) but untransformed scanpath length based on likelihood ratio tests. Detailed residual plots and Q-Q assessments are provided in Supplementary Figures S1–S3. Parameter estimates with 95% confidence intervals calculated using profile likelihood methods and standardized effect sizes (Cohen’s d for continuous outcomes, odds ratios for binary outcomes) are reported. Post hoc comparisons used Tukey’s HSD with familywise error correction (α = 0.05). Mediation analyses examined whether the effects of visual conditions on comprehension were mediated by changes in visual attention patterns. All degrees of freedom reported are exactly as output by the software packages.

Due to the remote nature of data collection, participants used varying hardware configurations that required standardization for meaningful comparison of eye-tracking metrics. WebGazer reports gaze coordinates in screen pixels, necessitating conversion to visual angles for interpretable saccade amplitudes and visual field measurements. At the beginning of each session, participants were prompted to provide their screen dimensions (width and height in inches) and display resolution (pixels). Viewing distance was estimated using a calibration procedure where participants positioned themselves so that a reference object (a credit card held at arm’s length) matched a displayed template, yielding an approximate viewing distance of 60 cm (range: 50–70 cm across participants). Visual angle conversion was calculated using the following formula: visual angle (degrees) = 2 × arctan(pixel_distance × screen_width_mm/(2 × screen_resolution_width × viewing_distance_mm)) × 180/π. This conversion assumes square pixels (equal horizontal and vertical pixel pitch). Potential anisotropy from non-square pixels was not assessed, though modern displays typically maintain pixel aspect ratios within 1% of unity, minimizing systematic bias in visual angle calculations. Here, screen width in mm = screen width in inches × 25.4. For participants who could not provide accurate screen dimensions (*n* = 23), we used device-independent relative units normalized to screen extent. Saccade amplitudes were expressed as proportions of total screen width, and scanpath lengths as proportions of total possible screen traversal distance.

The visual field deficit simulations were implemented using CSS-based overlay masks with carefully controlled luminance and contrast properties to ensure consistent visual impairment across participants while maintaining stimulus visibility. Central vision loss simulation: A circular mask with a 10° radius was positioned at screen center, filled with semi-transparent gray (RGB: 128, 128, 128) at 70% opacity (alpha = 0.7). This configuration reduced luminance in the affected region by approximately 30% while maintaining partial stimulus visibility to prevent complete information loss. Peripheral vision loss simulation: The visible area was restricted to a central circular region of 40° diameter, with the surrounding area masked using the same gray overlay (RGB: 128, 128, 128, alpha = 0.9) at 90% opacity, reducing peripheral luminance by approximately 90%. Scattered vision loss simulation: Multiple circular masks (each 3° diameter) were pseudo-randomly distributed across the display, covering approximately 15% of the total screen area. Each scatter point used identical gray overlay properties (RGB: 128, 128, 128, alpha = 0.8) at 80% opacity. All overlay masks were rendered in real-time using JavaScript Canvas API with anti-aliasing enabled to prevent sharp luminance transitions that might create artificial attention-capturing borders. The underlying economic stimuli maintained their original contrast ratios (minimum 7:1 for text elements, 4.5:1 for graphical elements) in non-masked regions to ensure accessibility standards compliance. Contrast ratios were measured using WebAIM’s Contrast Checker algorithm, which calculates luminance ratios according to WCAG 2.1 guidelines using the following formula: (L1 + 0.05)/(L2 + 0.05), where L1 is the relative luminance of the lighter color and L2 is the relative luminance of the darker color. All text elements (titles, axis labels, legends) were verified to meet or exceed 7:1 ratios, while graphical elements (chart bars, data points, grid lines) maintained minimum 4.5:1 ratios against their backgrounds.

## 4. Results

### 4.1. Demographic and Sample Characteristics

The final sample consisted of 227 participants after excluding 13 individuals due to poor eye-tracking data quality or technical difficulties. The mean age was 34.2 years (SD = 9.8), with 54% female participants. Educational backgrounds were diverse, with 68% holding bachelor’s degrees or higher. Participants reported an average of 4.3 years (SD = 3.1) of experience with financial or investment-related activities. The groups did not differ significantly on any demographic variables, confirming successful randomization. Following exclusions for missing eye-tracking data (>30% loss per trial), the effective trial counts per participant were as follows: Control: mean = 34.2 ± 2.8 trials (range: 28–36), Central Loss: mean = 33.8 ± 3.1 trials (range: 26–36), Peripheral Loss: mean = 34.5 ± 2.5 trials (range: 29–36), Scattered Loss: mean = 34.1 ± 2.9 trials (range: 27–36). There were no significant between-group differences in trial retention (F(3, 223) = 1.24, *p* = 0.296).

### 4.2. Eye-Tracking Results

Analysis of visual attention patterns revealed substantial differences across the four experimental conditions. Participants in the control condition demonstrated efficient scanning patterns with mean fixation durations of 287 ms (SD = 45 ms) and average saccade amplitudes of 5.2 degrees (SD = 1.3 degrees). In contrast, all three simulated deficit conditions showed altered oculomotor behaviors, with the magnitude of change varying by deficit type and information complexity.

The central vision loss group exhibited the most dramatic changes in viewing behavior. Mean fixation durations increased to 412 ms (SD = 78 ms), representing a 43.6% increase compared to controls (95% CI: 38.2–49.0%, d = 1.84). This group also showed significantly larger saccade amplitudes (M = 8.7 degrees, SD = 2.1 degrees) as participants attempted to use their simulated peripheral vision to extract information. Scanpath lengths were 68% longer than controls (95% CI: 59.3–76.7%, d = 1.92), indicating less efficient visual exploration. Particularly noteworthy was the tendency for these participants to adopt eccentric viewing strategies, positioning critical information away from their simulated central scotoma.

Participants with simulated peripheral vision loss demonstrated different but equally significant adaptations. Their fixation durations increased moderately to 341 ms (SD = 56 ms), while saccade amplitudes decreased to 3.1 degrees (SD = 0.9 degrees). This pattern suggests a more methodical, serial processing strategy necessitated by their restricted visual field. Total viewing time on areas of interest increased by 52% compared to controls (95% CI: 44.1–59.9%, d = 1.43), with particularly pronounced increases for information located in what would normally be peripheral regions of the display.

The scattered vision loss condition produced intermediate effects across most measures. Fixation durations averaged 358 ms (SD = 62 ms), with highly variable saccade patterns as participants attempted to navigate around simulated scotomas. These participants showed the highest revisiting rates for previously viewed areas, with 37% more refixations than controls, suggesting difficulty in maintaining spatial memory of information locations.

Table 1 presents a comprehensive summary of eye-tracking metrics across all experimental conditions and complexity levels. The data clearly illustrate the progressive deterioration in oculomotor efficiency as both visual deficit severity and stimulus complexity increase. Notably, the central vision loss group shows the most dramatic changes across all metrics, with fixation durations increasing from 387 ms to 441 ms as complexity increased from low to high, while their already elevated saccade amplitudes further increased from 8.2 to 9.1 degrees. The systematic reduction in AOI dwell time percentages across conditions reveals that participants with simulated deficits spent progressively less time examining critical information regions, with the central vision loss group spending only 54.2% of their time on relevant areas for high-complexity stimuli compared to 71.2% for controls.

The interaction between visual condition and information complexity proved particularly revealing. While control participants showed only modest increases in fixation duration as complexity increased (18% from low to high), the central vision loss group exhibited a 37% increase (95% CI: 29.4–44.6%, d = 1.18), suggesting that visual deficits compound the challenges posed by complex information. Area-of-interest analysis revealed that participants with simulated deficits spent significantly less time examining critical information regions, particularly axis labels and legends, which are essential for accurate interpretation of economic data. Overdispersion tests revealed adequate fit for fixation frequency Poisson models (deviance/df = 1.12–1.34 across conditions). Log-transformed fixation duration models showed improved residual distributions (Supplementary Figure S1) and were used for all analyses.

Detailed error analysis revealed deficit-specific patterns with direct implications for accommodation strategies. Participants with central vision loss showed particular difficulty with precise numerical extraction, achieving only 43% accuracy on questions requiring exact values compared to 89% for controls. This group made 57% of their errors on numerical tasks and 34% on trend identification. Peripheral vision loss most strongly affected spatial relationship comprehension, with 45% of errors involving questions about relationships between spatially separated elements and 28% involving comparative analyses across multiple data points. Scattered vision loss produced more distributed impairment patterns, with 38% error rates across all question categories and no specific vulnerability pattern. Cognitive load dimension analysis revealed that mental demand showed the largest between-group effect sizes (d = 2.31 for central vision loss), while frustration levels correlated most strongly with comprehension failures (r = −0.72). Effort ratings predicted task abandonment behaviors, with 23% of central vision loss participants requiring additional breaks or expressing desire to discontinue the task. Time-course analysis showed that group differences emerged early and persisted: during the first 10 s, all deficit groups required 40–60% longer orientation periods; in the middle phase (10–20 s), the central vision loss group showed 73% more backtracking behaviors; during the final phase (20–30 s), the peripheral vision loss group spent 52% more time on information verification.

### 4.3. Survey-Based Results

Cognitive load measurements using the NASA-TLX revealed significant group differences across all six dimensions. The central vision loss group reported the highest overall cognitive load scores (M = 72.3, SD = 11.2), followed by scattered loss (M = 65.8, SD = 10.4), peripheral loss (M = 61.2, SD = 9.8), and control (M = 41.6, SD = 8.3) groups. Mental demand and effort subscales showed the largest group differences, with central vision loss participants rating mental demand 84% higher than controls (95% CI: 71.2–96.8%, d = 2.31).

The frustration dimension of the NASA-TLX yielded particularly interesting results. While control participants reported minimal frustration even with high-complexity stimuli (M = 28.1 on the 0–100 scale), participants with simulated central vision loss reported frustration levels averaging 72.4, with several participants commenting in post-task feedback about the difficulty of extracting specific numerical values from charts and graphs.

Economic comprehension was analyzed at the trial level using logistic mixed-effects models. The base model revealed substantial between-participant variance (ICC = 0.18, indicating 18% of variance attributable to participant-level differences). Marginal R^2^ = 0.24 and conditional R^2^ = 0.38, indicating that visual condition and complexity explained 24% of variance, with an additional 14% explained by participant random effects. Primary logistic model results showed significant main effects of visual condition (likelihood ratio test: χ^2^(3) = 89.4, *p* < 0.001). Compared to controls, the odds of correct responses were substantially reduced across all deficit conditions: central vision loss (OR = 0.31, 95% CI [0.24, 0.39], *p* < 0.001), peripheral vision loss (OR = 0.52, 95% CI [0.41, 0.66], *p* < 0.001), and scattered vision loss (OR = 0.44, 95% CI [0.35, 0.56], *p* < 0.001). Converting to predicted probabilities for interpretability revealed that control participants achieved 87.3% accuracy (95% CI [84.1%, 90.1%]), while those with central loss, peripheral loss, and scattered loss achieved 61.2% (95% CI [56.8%, 65.4%]), 74.6% (95% CI [70.9%, 78.0%]), and 69.8% (95% CI [66.0%, 73.4%]), respectively. Secondary linear probability models confirmed these patterns, with central vision loss showing a −26.1 percentage point reduction (95% CI [−31.4, −20.8], *p* < 0.001) compared to controls. The conclusions remain unchanged whether logistic or linear specifications were used.

The relationship between objective eye-tracking measures and subjective experiences was examined through correlation analyses. Longer fixation durations correlated positively with reported cognitive load (r = 0.68, *p* < 0.001) and negatively with comprehension accuracy (r = −0.54, *p* < 0.001). Similarly, increased scanpath length showed strong associations with both higher cognitive load (r = 0.71, *p* < 0.001) and lower comprehension (r = −0.62, *p* < 0.001).

Table 2 provides a detailed breakdown of cognitive load dimensions and their relationship with comprehension performance across visual conditions. The data reveal that mental demand and effort dimensions showed the largest disparities between groups, with central vision loss participants rating these dimensions 78.1 and 81.2, respectively, nearly double the control group values. The frustration scores particularly highlight the subjective difficulty experienced, increasing from 28.1 in controls to 72.4 in the central vision loss group. These elevated cognitive load scores correspond inversely with comprehension accuracy, supporting the hypothesis that increased perceptual processing demands consume cognitive resources that would otherwise be available for information comprehension and integration.

Sensitivity analyses controlling for device characteristics and tracking quality confirmed the robustness of primary findings, with effect sizes remaining large despite slight attenuation when accounting for these potential confounds (see Supplementary Table S1).

Table 3 presents the results from the primary logistic mixed-effects model analysis of comprehension accuracy. The odds ratios quantify the multiplicative change in odds of answering correctly for each visual deficit condition relative to controls. For example, participants with central vision loss had 0.31 times the odds (or 69% lower odds) of correct responses compared to controls. The interaction terms reveal that the negative effects of visual deficits were amplified for high-complexity stimuli, with the Central × High interaction (OR = 0.64) indicating an additional 36% reduction in odds beyond the main effects. The predicted probabilities in the rightmost column translate these odds ratios into more interpretable accuracy percentages, showing the substantial performance gaps between conditions.

### 4.4. Relationship Between Visual Attention and Subjective Measures

Multilevel mediation analysis revealed that altered fixation patterns mediated comprehension deficits across all visual conditions. Results are presented in Table 4. Monte Carlo confidence intervals accounted for the multilevel data structure. Sensitivity analysis assuming unmeasured confounder correlation ρ = 0.2 yielded similar conclusions (central: 43% mediated, peripheral: 31%, scattered: 38%).

Sequential analysis of viewing patterns revealed qualitative differences in information extraction strategies across groups. Control participants typically followed a systematic pattern, beginning with titles and axis labels before examining data points. In contrast, participants with simulated deficits showed more erratic patterns, often returning multiple times to reference information they had difficulty encoding on initial viewing. The central vision loss group showed particularly fragmented viewing patterns, with frequent transitions between distant screen regions as they attempted to position information within their functional peripheral vision.

**Figure 1:**
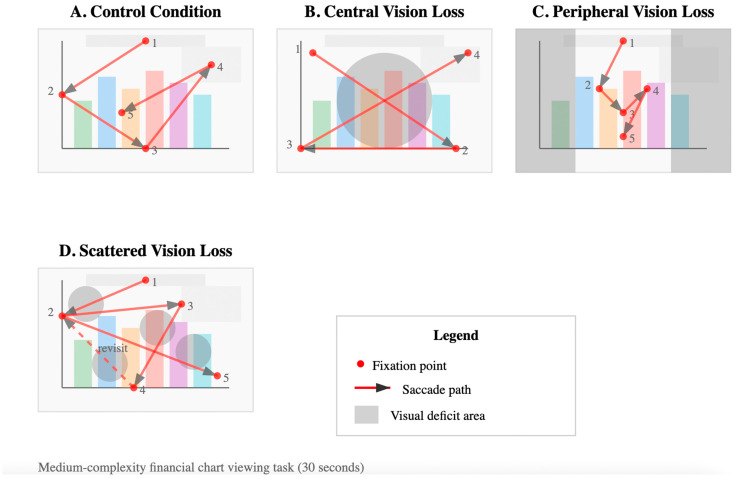
Representative scanpath patterns across visual conditions. Red circles mark fixation locations, with numbers (1–5) indicating the temporal sequence of fixations. Red solid lines with arrows denote saccade paths (direction of eye movements between successive fixations).

Figure 1 shows representative scanpath patterns across visual conditions during viewing of a medium-complexity financial chart. Four panels illustrate typical eye movement behaviors for each experimental condition over a 30 s viewing period. Panel A (Control Condition) demonstrates efficient, systematic scanning with orderly progression from title to axes to data points to legend, characteristic of normal visual processing. Panel B (Central Vision Loss) shows large, sweeping eye movements avoiding the central region, with participants utilizing peripheral vision to compensate for the simulated central scotoma (gray circle, 10° radius). Panel C (Peripheral Vision Loss) displays concentrated scanning patterns restricted to the central visual field, with small-amplitude saccades and dense fixation clusters within the preserved central 40° of vision (gray bars indicate non-visible peripheral regions). Panel D (Scattered Vision Loss) reveals irregular, fragmented scanning patterns with frequent backtracking and refixations as participants navigate around multiple simulated scotomas (gray circles). Red circles indicate fixation locations, with numbers showing temporal sequence. Red lines represent saccade paths, with arrows indicating direction of movement. Dashed lines indicate revisits to previously viewed areas. These distinct oculomotor strategies demonstrate how different types of visual field deficits lead to specific compensatory viewing behaviors, which may explain the observed differences in comprehension accuracy and cognitive load across conditions.

Analysis of time-course data revealed that group differences emerged early in viewing and persisted throughout the 30 s presentation period. Participants with simulated deficits required an average of 8.7 s to complete their first systematic scan of the stimulus, compared to 4.2 s for controls. This initial orientation period appeared critical, as participants who took longer to establish viewing strategies showed poorer comprehension regardless of total viewing time.

The complexity manipulation interacted significantly with visual condition across all dependent measures. While control participants showed only modest decrements in performance as complexity increased, the visual deficit groups showed accelerating impairment. For high-complexity stimuli, the comprehension gap between control and central vision loss groups widened to 34 percentage points, compared to only 19 percentage points for low-complexity stimuli. This pattern suggests that visual impairments not only affect basic information extraction but also limit the cognitive resources available for integrating complex information.

Post hoc analyses examining specific types of comprehension errors revealed systematic patterns. Participants with central vision loss were particularly prone to errors involving precise numerical values, achieving only 43% accuracy on questions requiring exact number identification compared to 89% for controls. Peripheral vision loss most strongly affected questions about relationships between spatially separated elements, while scattered vision loss produced more general decrements across all question types.

Our results also provide clear answers to the three primary research questions posed in this study. Research Question 1: How do different types of simulated visual field deficits affect eye movement patterns when economic information is viewed? Hypothesis 1a was supported: Participants with simulated central vision loss exhibited significantly longer fixation durations (412 ms vs. 287 ms for controls, d = 1.84) and larger saccade amplitudes (8.7° vs. 5.2° for controls, d = 1.92), confirming compensatory peripheral viewing strategies. Hypothesis 1b was supported: Participants with simulated peripheral vision loss showed significantly shorter saccade amplitudes (3.1° vs. 5.2° for controls, d = −1.67) and more concentrated fixation patterns within the central visual field, as evidenced by reduced scanpath lengths and higher fixation density in central AOIs. Hypothesis 1c was supported: Participants with scattered vision loss demonstrated more erratic scanning patterns with 37% more refixations than controls and intermediate scanpath lengths (0.50–0.67 normalized units), indicating difficulty maintaining spatial memory of information locations. Research Question 2: Do visual field deficits interact with information complexity to affect comprehension of economic information? Hypothesis 2 was strongly supported: The interaction between visual condition and complexity was significant. Testing was performed using a linear mixed-effects model (lme4::lmer, R 4.3.2) with the following specification: Comprehension ~ Condition × Complexity + (1|Participant). A likelihood ratio test comparing this full model against a reduced model without the interaction term yielded χ^2^(6) = 48.92, *p* < 0.001. Post hoc contrasts computed with emmeans using Kenward–Roger approximation revealed that the complexity effect differed significantly between visual conditions (Central vs. Control × Complexity: t(6751) = −5.87, *p* < 0.001, df as computed by pbkrtest; Peripheral vs. Control × Complexity: t(6750) = −3.42, *p* = 0.003; Scattered vs. Control × Complexity: t(6750) = −4.21, *p* < 0.001). While control participants showed only modest decrements with increasing complexity (18% decrease from low to high), the central vision loss group exhibited a 37% decrease (95% CI: 29.4–44.6%, d = 1.18), confirming a multiplicative rather than additive effect. The comprehension gap between control and central vision loss groups widened from 19 percentage points for low-complexity stimuli to 34 percentage points for high-complexity stimuli. Research Question 3: What is the relationship between altered visual attention patterns and subjective cognitive load in individuals with simulated visual field deficits? Hypothesis 3a was supported: All visual deficit groups reported significantly higher cognitive load than controls, with central vision loss showing the largest effect (M = 72.3 vs. 41.6 for controls, d = 2.31). Mental demand ratings were 84% higher for central vision loss participants (95% CI: 71.2–96.8%). Hypothesis 3b was supported: Mediation analysis confirmed that altered visual attention patterns partially mediated the relationship between visual condition and comprehension. For central vision loss, 47% of the comprehension deficit was mediated through increased fixation durations and reduced time on critical AOIs (β = −0.31, 95% CI: −0.47, −0.15). Strong correlations between objective eye-tracking measures and subjective experiences validated this relationship (fixation duration–cognitive load: r = 0.68; scanpath length–cognitive load: r = 0.71). These findings collectively demonstrate that simulated visual field deficits systematically alter economic information processing through both direct perceptual limitations and compensatory attention strategies that impose additional cognitive demands.

### 4.5. Practical Implementation Implications

Our findings provide specific guidance for designing accessible financial information systems and accommodating individuals with visual impairments in economic contexts. For central vision loss accommodations, financial interfaces should implement peripheral information highlighting with high-contrast borders, provide adjustable content positioning allowing users to move critical information away from central screen areas, offer audio narration for precise numerical values that are difficult to extract peripherally, and design layouts with important information distributed across the visual field rather than centrally concentrated. For peripheral vision loss accommodations, systems should maximize central information density while maintaining readability, implement scrollable content windows that fit within the central 40° of vision, provide detailed zoom capabilities for fine-grained analysis, and design compact summary views that present key information within restricted visual fields. For scattered vision loss accommodations, interfaces should implement customizable overlay options allowing users to mask problematic screen regions, provide alternative information presentation formats (text versus graphical), and design adaptive systems that can relocate content based on user-identified problem areas. Professional practice guidelines are as follows: our results suggest that financial advisors working with visually impaired clients should allow 2–3 times longer for document review (based on our observed 68% increase in processing time under 30 s viewing constraints; self-paced viewing may show different patterns), provide information in multiple formats simultaneously, recognize that cognitive fatigue occurs more rapidly (84% higher mental demand), and implement structured information presentation following systematic scanning patterns. Regulatory considerations are as follows: Current accessibility standards may be insufficient for complex economic information. Our findings suggest that simple magnification approaches may be inadequate and that format modification is necessary; one-size-fits-all approaches are inappropriate, and deficit-specific accommodations are needed; processing time allowances should be increased by 50–70% for complex financial documents; and alternative assessment methods may be necessary for financial literacy evaluation.

## 5. Discussion

Our study provides compelling evidence that simulated ophthalmic visual field deficits significantly impair the processing of economic information, with distinct patterns of impairment associated with different types of visual loss. The observed alterations in eye movement patterns, increased cognitive load, and reduced comprehension accuracy have important implications for understanding how individuals with visual impairments navigate economic decisions in daily life. These findings extend previous research on visual attention in decision-making by demonstrating that the relationship between visual processing and economic cognition is fundamentally altered when visual field deficits are present.

The dramatic changes in oculomotor behavior observed across all three deficit conditions reflect adaptive strategies that, while potentially beneficial for general visual exploration, appear maladaptive for processing complex economic information. Participants with simulated central vision loss showed the most severe impairments, with fixation durations increasing by over 40% and comprehension accuracy dropping markedly for complex stimuli. This finding aligns with research by Crossland et al. [28], who documented similar compensatory eye movement patterns in patients with macular degeneration during reading tasks. However, our results suggest that these adaptations may be particularly problematic when processing graphical economic information, where spatial relationships between data points are crucial for accurate interpretation.

The interaction between visual deficit type and information complexity revealed in our study has not been previously documented in the context of economic decision-making. While all participants showed some performance degradation with increasing complexity, the accelerated impairment observed in the visual deficit groups suggests a multiplicative rather than additive effect. This pattern is consistent with cognitive load theory, which predicts that when perceptual processing demands increase, fewer cognitive resources remain available for higher-level comprehension and integration tasks. The particularly steep decline in performance for central vision loss participants viewing high-complexity stimuli indicates that there may be a threshold beyond which compensatory strategies fail entirely.

Our finding that peripheral vision loss produced distinct but less severe impairments than central vision loss has important theoretical implications. The visual system’s organization, with high-acuity foveal vision specialized for detail extraction and lower-acuity peripheral vision specialized for spatial context and motion detection, suggests that different types of economic information may be differentially affected by various visual field deficits. A study [29] has demonstrated that peripheral vision contributes significantly to scene gist extraction and spatial memory formation, functions that our results suggest are crucial for maintaining mental models of complex economic displays. The relatively preserved performance of peripheral vision loss participants on simple stimuli but marked impairment on complex displays supports this interpretation.

The strong correlations between objective eye-tracking measures and subjective cognitive load ratings validate the use of eye movements as indicators of processing difficulty in visually impaired populations. This relationship has been explored in other domains, with a study [30] showing that fixation duration increases and saccadic efficiency decreases are reliable markers of cognitive load during complex visual tasks. Our extension of these findings to economic information processing suggests that eye-tracking methodologies could be valuable for assessing the accessibility of financial interfaces and documents for individuals with visual impairments.

The practical implications of our findings are substantial. Current standards for financial document design rarely consider the specific needs of individuals with different types of visual field deficits. Our results suggest that one-size-fits-all approaches to accessibility may be insufficient, as the optimal information presentation format likely depends on the specific nature of an individual’s visual impairment. For instance, while magnification might help those with central vision loss, our data suggest that it could potentially harm those with peripheral vision loss by further restricting the amount of information visible within their limited visual field. This finding echoes concerns raised by Lee et al. in [31] about the need for personalized approaches to visual accessibility.

The use of webcam-based eye tracking through MTurk in our study demonstrates the feasibility of conducting large-scale vision research outside traditional laboratory settings. While some precision is sacrificed compared to laboratory-grade eye trackers, the ability to recruit diverse participants and study real-world viewing conditions may outweigh these limitations for many research questions. Future studies could examine whether these simulation-based findings extend to individuals with actual visual impairments, though additional validation of webcam-based tracking in clinical populations would be necessary.

Several limitations of our study warrant consideration. First, our participant sample consisted entirely of individuals with normal vision experiencing simulated deficits, fundamentally limiting the generalizability to clinical populations. Patients with actual visual field deficits develop sophisticated compensatory strategies over months or years of adaptation, including preferred retinal locus development in macular degeneration patients, systematic scanning patterns that maximize information extraction from preserved visual fields, and cognitive adaptations that redistribute processing load across sensory modalities. The oculomotor behaviors we observed likely represent immediate, suboptimal responses to novel visual constraints rather than the refined compensatory strategies characteristic of adapted patients. This limitation is particularly important given that research by Crossland et al. [28] demonstrates that patients with macular degeneration develop highly individualized eccentric viewing strategies that substantially improve their functional performance over time, suggesting our findings may overestimate the true functional impact of visual impairments on economic information processing. Second, while our simulations approximated scotoma patterns and field loss configurations, they cannot replicate the full complexity of clinical visual impairments. Real ophthalmic conditions frequently involve additional factors including reduced contrast sensitivity (particularly problematic for reading financial text), altered color perception (affecting interpretation of color-coded financial graphics), photophobia (limiting viewing comfort and duration), metamorphopsia or visual distortions (affecting spatial relationships in charts), and fluctuating vision quality throughout the day (creating inconsistent information processing capabilities). These factors may interact with attention allocation and cognitive load in ways not captured by our transparency-based simulations. Furthermore, the overlay-based approach creates uniform luminance reduction rather than the complete information loss characteristic of true scotomas, potentially allowing participants to extract some information from ‘impaired’ regions that would be completely unavailable to actual patients. Third, our fixed 30 s viewing paradigm, while ensuring experimental control and sufficient data for eye-tracking analysis, differs substantially from real-world economic information processing contexts. In naturalistic financial decision-making situations, individuals can revisit information multiple times across different sessions, spend varying amounts of time based on personal decision complexity and stakes, integrate information from multiple sources or documents, and take breaks to reduce cognitive fatigue. The imposed time constraint may have particularly disadvantaged participants with simulated deficits who require additional processing time to extract equivalent information, potentially inflating the observed performance differences between groups. Fourth, the remote data collection necessitated relying on participant-reported screen dimensions and estimated viewing distances, introducing potential measurement error. Approximately 10% of participants could not provide accurate device specifications, requiring the use of device-independent relative metrics. While this approach enables broader participation, it limits the precision of absolute visual angle measurements and may introduce variance in effect size estimates. Future studies should consider implementing automated device detection or providing standardized viewing setups to improve measurement consistency. Fifth, the visual overlay masks may have degraded WebGazer precision by altering contrast patterns, particularly for central vision loss simulations. We did not include control conditions with equivalent non-informative overlays to quantify this potential confound. Last but not least, our complexity manipulation confounded multiple dimensions (element count, visual annotations, legend detail), making it difficult to isolate specific sources of difficulty. Future studies should use factorial designs to separate these factors and analyze stimulus types (charts, tables, plots) independently.

Future research should prioritize validation studies with actual clinical populations to determine whether our simulation-based findings generalize to individuals with real visual impairments and established compensatory strategies. Such studies should incorporate longitudinal components to examine how economic information processing abilities evolve during the adaptation period following vision loss, potentially revealing recovery of function not captured in our acute simulation paradigm. Additionally, investigation of variable viewing time paradigms and decision-based tasks would better approximate real-world economic contexts where individuals control their information exposure duration and can revisit critical information. The development of adaptive display technologies that adjust information presentation based on an individual’s specific visual capabilities represents a promising direction. Additionally, investigating how other sensory modalities, such as auditory or tactile feedback, might complement visual information processing for economic decision-making could lead to more inclusive design solutions.

Ultimately, this research represents an important first step toward understanding how visual impairments affect economic cognition, while highlighting the critical need for validation studies with clinical populations to ensure these findings translate to real-world applications.

## Figures and Tables

**Table 1:** Eye-tracking metrics by visual condition and complexity level. (Statistical note: All inferential tests performed using linear mixed-effects models (lme4::lmer, version 1.1-35, R 4.3.2) with REML estimation. Model: Metric ~ Condition × Complexity + (1|Participant). Pairwise comparisons computed using emmeans (version 1.8.9) with Kenward–Roger degrees of freedom (pbkrtest version 0.5-0.2).)

Visual Condition	Complexity	Fixation Duration (ms)	Saccade Amplitude (deg)	Scanpath Length (norm.)	AOI Dwell Time (%)
Control	Low	265.00 ± 41.00	4.80 ± 1.10	0.42 ± 0.07	82.30 ± 7.20
	Medium	284.00 ± 43.00	5.30 ± 1.30	0.50 ± 0.08	78.60 ± 8.10
	High	312.00 ± 48.00	5.50 ± 1.40	0.57 ± 0.10	71.20 ± 9.30
Central Loss	Low	387.00 ± 71.00	8.20 ± 1.90	0.68 ± 0.12	68.70 ± 11.20
	Medium	409.00 ± 76.00	8.70 ± 2.10	0.81 ± 0.15	61.30 ± 12.80
	High	441.00 ± 84.00	9.10 ± 2.30	0.97 ± 0.17	54.20 ± 14.10
Peripheral Loss	Low	318.00 ± 52.00	2.90 ± 0.80	0.45 ± 0.07	75.60 ± 8.90
	Medium	339.00 ± 55.00	3.10 ± 0.90	0.53 ± 0.09	69.80 ± 10.20
	High	366.00 ± 61.00	3.30 ± 1.00	0.62 ± 0.11	62.10 ± 11.70
Scattered Loss	Low	334.00 ± 58.00	4.10 ± 1.20	0.50 ± 0.09	71.20 ± 9.80
	Medium	356.00 ± 61.00	4.50 ± 1.40	0.57 ± 0.10	65.40 ± 11.10
	High	385.00 ± 67.00	4.80 ± 1.60	0.67 ± 0.12	58.70 ± 12.40

Note: Saccade amplitudes reported in visual angle degrees for participants with available device specifications (*n* = 204). Screen width proportions for remaining participants (*n* = 23) are provided in Supplementary Table S2. Scanpath lengths normalized to maximum possible screen traversal distance (0–1 scale). AOI = Area of Interest.

**Table 2:** Cognitive load and comprehension measures by visual condition.

Visual Condition	NASA-TLX Total	Mental Demand	Physical Demand	Temporal Demand	Performance	Effort	Frustration	Comprehension (%)
Control	41.6 ± 8.3	42.3 ± 9.1	23.4 ± 7.2	38.7 ± 8.9	35.2 ± 8.1	44.6 ± 9.7	28.1 ± 8.4	87.3 ± 9.2
Central Loss	72.3 ± 11.2	78.1 ± 10.3	34.6 ± 9.8	67.3 ± 11.2	69.8 ± 10.7	81.2 ± 11.9	72.4 ± 12.3	61.2 ± 14.8
Peripheral Loss	61.2 ± 9.8	63.4 ± 9.7	29.8 ± 8.3	56.2 ± 10.1	58.7 ± 9.4	68.3 ± 10.2	54.6 ± 10.8	74.6 ± 11.3
Scattered Loss	65.8 ± 10.4	68.9 ± 10.1	31.2 ± 8.7	61.4 ± 10.8	63.2 ± 9.9	74.6 ± 10.9	61.3 ± 11.4	69.8 ± 12.7

**Table 3:** Logistic mixed-effects model results for comprehension accuracy.

Visual Condition	Odds Ratio	95% CI	z-Value	*p*-Value	Predicted Probability (%)
Control (Reference)	1.00	-	-	-	87.3 [84.1, 90.1]
Central Loss	0.31	[0.24, 0.39]	−8.92	<0.001	61.2 [56.8, 65.4]
Peripheral Loss	0.52	[0.41, 0.66]	−5.23	<0.001	74.6 [70.9, 78.0]
Scattered Loss	0.44	[0.35, 0.56]	−6.47	<0.001	69.8 [66.0, 73.4]
**Complexity Level**					
Low (Reference)	1.00	-	-	-	82.4 [79.8, 84.8]
Medium	0.76	[0.68, 0.85]	−4.82	<0.001	76.3 [73.5, 78.9]
High	0.58	[0.52, 0.65]	−8.13	<0.001	69.1 [66.2, 71.9]
**Interaction Effects (Condition × Complexity)**					
Central × Medium	0.82	[0.67, 1.01]	−1.87	0.062	-
Central × High	0.64	[0.52, 0.79]	−4.21	<0.001	-
Peripheral × Medium	0.91	[0.74, 1.11]	−0.94	0.347	-
Peripheral × High	0.78	[0.64, 0.96]	−2.35	0.019	-
Scattered × Medium	0.86	[0.70, 1.05]	−1.48	0.139	-
Scattered × High	0.71	[0.58, 0.87]	−3.24	0.001	-

Model fit statistics: Marginal R^2^ = 0.24, Conditional R^2^ = 0.38, ICC = 0.18. Note: Model specification: glmer(Accuracy ~ Condition × Complexity + (1|Participant), family = binomial, data = trial_data). N = 8172 trials from 227 participants. Odds ratios < 1 indicate reduced odds of correct response relative to reference category.

**Table 4:** Multilevel mediation analysis results.

Condition	Total Effect β (95% CI)	Direct Effect β (95% CI)	Indirect Effect β (95% CI)	% Mediated
Central	−0.66 (−0.82, −0.50)	−0.35 (−0.48, −0.22)	−0.31 (−0.47, −0.15)	47%
Peripheral	−0.38 (−0.51, −0.25)	−0.25 (−0.36, −0.14)	−0.13 (−0.23, −0.03)	34%
Scattered	−0.44 (−0.57, −0.31)	−0.26 (−0.37, −0.15)	−0.18 (−0.31, −0.05)	41%

Note: Monte Carlo 95% confidence intervals based on 10,000 simulations.

## Data Availability

The dataset is available on request from the authors. Raw gaze coordinates with high-resolution timestamps cannot be made publicly available because, in combination with device metadata (screen size/resolution), they could permit re-identification of participants and violate our ethics approval. To balance participant privacy and reproducibility, we make the following available to qualified researchers under a Data Use Agreement (DUA): (a) the full analysis code and computational environment specification, (b) the complete set of experimental stimuli and task scripts, and (c) a derived, de-identified trial-level dataset that includes all variables used in the analyses (attention metrics expressed in degrees or screen-normalized units, AOI measures, NASA-TLX scores, comprehension outcomes, condition/complexity indicators, and device-quality covariates), but we exclude raw gaze coordinates, raw video, and direct device identifiers. Requests will be evaluated by the authors in line with the consent and ethics approvals; approved requests will receive an encrypted transfer within 10 business days. Researchers can contact the corresponding author to initiate the DUA process.

## Supplementary Materials

The following supporting information can be downloaded at https://www.mdpi.com/article/10.3390/jemr18050050/s1: Figure S1: Model diagnostics; Figure S2: Overdispersion assessment; Figure S3: Model comparison; Table S1: Sensitivity analyses controlling for device characteristics and tracking quality; Table S2: Saccade amplitudes in screen width proportions for participants with unavailable device specifications (*n* = 23); Table S3: NASA-TLX subscale distributions by visual condition.
