# Supplementary material for: Visual Attention to Economic Information in Simulated Ophthalmic Deficits: A Remote Eye-Tracking Study
Source: J Eye Mov Res. 2025 Oct 2;18(5):50. doi: 10.3390/jemr18050050 (PMC12565663; doi:10.3390/jemr18050050)
Supplement: Supplementary file 1 [file jemr-18-00050-s001.zip › jemr-3815087-supplementary.pdf]

Supplementary Materials

Visual Attention to Economic Information in Simulated Ophthalmic Deficits: A Remote Eye-Tracking Study

Cansu Yuksel Elgin [1,*] and Ceyhun Elgin [2]

[1] Department of Ophthalmology, Istanbul University-Cerrahpasa, Istanbul 34098, Türkiye
[2] Department of Economics, Bogazici University, Istanbul 34342, Türkiye; ceyhun.elgin@bogazici.edu.tr
* Correspondence: cansu.elgin@iuc.edu.tr; Tel.: +90-537-503-35-05

Supplementary Table S1. Sensitivity analyses controlling for device characteristics and tracking quality.

Outcome Variable	Main Effect	Baseline Model	+ Device Controls	+ Tracking Quality	Full Model
**Fixation Duration**					
	Central vs. Control β (95% CI)	124.2 (98.3, 150.1)***	119.7 (94.1, 145.3)***	116.8 (91.5, 142.1)***	114.3 (89.2, 139.4)***
	d	1.84	1.79	1.75	1.72
	Peripheral vs. Control β (95% CI)	54.1 (28.7, 79.5)***	51.3 (26.2, 76.4)***	49.8 (24.9, 74.7)***	47.9 (23.1, 72.7)***
	d	1.21	1.15	1.12	1.08
	Scattered vs. Control β (95% CI)	71.2 (46.1, 96.3)***	68.7 (43.8, 93.6)***	66.4 (41.7, 91.1)***	64.8 (40.2, 89.4)***
	d	1.43	1.38	1.34	1.31
**Saccade Amplitude**					
	Central vs. Control β (95% CI)	3.9 (3.2, 4.6)***	3.7 (3.0, 4.4)***	3.6 (2.9, 4.3)***	3.5 (2.8, 4.2)***
	d	1.92	1.85	1.81	1.77
	Peripheral vs. Control β (95% CI)	-2.1 (-2.7, -1.5)***	-2.0 (-2.6, -1.4)***	-1.9 (-2.5, -1.3)***	-1.8 (-2.4, -1.2)***
	d	-1.67	-1.62	-1.58	-1.54
**Comprehension Accuracy (Linear Probability Model)**					
	Central vs. Control β (95% CI)	-26.1 (-31.4, -20.8)***	-24.8 (-30.1, -19.5)***	-23.9 (-29.2, -18.6)***	-23.1 (-28.4, -17.8)***
	d	-2.15	-2.07	-2.01	-1.96
	Peripheral vs. Control β (95% CI)	-12.7 (-17.8, -7.6)***	-11.9 (-16.9, -6.9)***	-11.3 (-16.3, -6.3)***	-10.8 (-15.7, -5.9)***
	d	-1.28	-1.22	-1.18	-1.14

Outcome Variable	Main Effect	Baseline Model	+ Device Controls	+ Tracking Quality	Full Model
	Scattered vs. Control β (95% CI)	-17.5 (-22.6, -12.4)***	-16.8 (-21.8, -11.8)***	-16.2 (-21.2, -11.2)***	-15.7 (-20.6, -10.8)***
	d	-1.52	-1.47	-1.43	-1.39
**Comprehension Accuracy (Logistic Model)**					
	Central vs. Control OR (95% CI)	0.31 (0.24, 0.39)***	0.33 (0.26, 0.42)***	0.34 (0.27, 0.43)***	0.35 (0.28, 0.44)***
	Peripheral vs. Control OR (95% CI)	0.52 (0.41, 0.66)***	0.54 (0.43, 0.68)***	0.55 (0.44, 0.70)***	0.57 (0.45, 0.72)***
	Scattered vs. Control OR (95% CI)	0.44 (0.35, 0.56)***	0.46 (0.36, 0.58)***	0.47 (0.37, 0.59)***	0.48 (0.38, 0.61)***
**NASA-TLX Total**					
	Central vs. Control β (95% CI)	30.7 (24.2, 37.2)***	29.1 (22.7, 35.5)***	28.4 (22.1, 34.7)***	27.6 (21.4, 33.8)***
	d	2.31	2.23	2.18	2.12
	Peripheral vs. Control β (95% CI)	19.6 (13.4, 25.8)***	18.7 (12.6, 24.8)***	18.1 (12.1, 24.1)***	17.4 (11.5, 23.3)***
	d	1.76	1.69	1.64	1.58
	Scattered vs. Control β (95% CI)	24.2 (18.0, 30.4)***	23.1 (17.0, 29.2)***	22.5 (16.5, 28.5)***	21.8 (15.9, 27.7)***
	d	1.98	1.91	1.87	1.82

Note: Baseline Model = Visual Condition × Complexity + (1 + Complexity | Participant). Device Controls = screen size, resolution, viewing distance. Tracking Quality = calibration accuracy, drift frequency. Full Model includes all covariates. β = unstandardized coefficient; d = Cohen's d effect size; OR = odds ratio; CI = confidence interval. ***p < 0.001.

Statistical implementation: All continuous outcomes fitted using lme4::lmer() in R 4.3.2 with REML estimation. Comprehension accuracy analyzed using both linear probability models (lmer) for interpretability and logistic models (glmer with binomial family) as the primary specification. Kenward–Roger degrees of freedom approximation via pbkrtest. Each model specification and degrees of freedom are as output directly by the software. Baseline Model: Outcome.

Supplementary Table S2. Saccade amplitudes in screen width proportions for participants with unavailable device specifications (n=23).

Visual Condition	Complexity	Saccade Amplitude (screen width proportions)
**Control**	Low	0.12 ± 0.03
	Medium	0.13 ± 0.03

Visual Condition	Complexity	Saccade Amplitude (screen width proportions)
	High	0.14 ± 0.04
**Central Loss**	Low	0.20 ± 0.05
	Medium	0.22 ± 0.05
	High	0.23 ± 0.06
**Peripheral Loss**	Low	0.07 ± 0.02
	Medium	0.08 ± 0.02
	High	0.08 ± 0.03
**Scattered Loss**	Low	0.10 ± 0.03
	Medium	0.11 ± 0.04
	High	0.12 ± 0.04

Note: This table provides saccade amplitude data for the subset of participants (n=23) who could not provide accurate screen dimension specifications. Values are expressed as proportions of total screen width. For the majority of participants (n=204), saccade amplitudes in visual angle degrees are reported in Table 1.

Supplementary Table S3. NASA-TLX subscale distributions by visual condition.

Visual Condition	Mental Demand	Physical Demand	Temporal Demand	Performance	Effort	Frustration
**Control**						
Mean ± SD	42.3 ± 9.1	23.4 ± 7.2	38.7 ± 8.9	35.2 ± 8.1	44.6 ± 9.7	28.1 ± 8.4
Median [IQR]	41.0 [36.0-48.0]	22.0 [18.0-28.0]	37.0 [32.0-45.0]	34.0 [29.0-41.0]	43.0 [38.0-51.0]	26.0 [22.0-33.0]
Min–Max	18-67	8-42	16-63	12-58	19-71	8-52
**Central Loss**						
Mean ± SD	78.1 ± 10.3	34.6 ± 9.8	67.3 ± 11.2	69.8 ± 10.7	81.2 ± 11.9	72.4 ± 12.3
Median [IQR]	79.0 [71.0-86.0]	33.0 [27.0-42.0]	68.0 [59.0-76.0]	71.0 [62.0-78.0]	82.0 [73.0-90.0]	74.0 [63.0-82.0]
Min–Max	48-98	12-59	32-91	38-92	45-100	34-96
**Peripheral Loss**						
Mean ± SD	63.4 ± 9.7	29.8 ± 8.3	56.2 ± 10.1	58.7 ± 9.4	68.3 ± 10.2	54.6 ± 10.8
Median [IQR]	64.0 [57.0-71.0]	29.0 [24.0-36.0]	57.0 [49.0-64.0]	59.0 [52.0-66.0]	69.0 [61.0-76.0]	55.0 [47.0-63.0]
Min–Max	36-86	11-49	28-78	31-81	38-91	24-79
**Scattered Loss**						
Mean ± SD	68.9 ± 10.1	31.2 ± 8.7	61.4 ± 10.8	63.2 ± 9.9	74.6 ± 10.9	61.3 ± 11.4
Median [IQR]	69.0 [62.0-77.0]	31.0 [25.0-38.0]	62.0 [53.0-70.0]	64.0 [56.0-71.0]	75.0 [67.0-83.0]	62.0 [53.0-70.0]

Visual Condition	Mental Demand	Physical Demand	Temporal Demand	Performance	Effort	Frustration
Min–Max	41-92	13-54	31-84	35-86	42-96	28-87

Note: All subscales measured on 0-100 scales. IQR = interquartile range (25th-75th percentiles). Higher scores indicate greater perceived load/demand except for Performance, where higher scores indicate better perceived performance. Raw TLX scoring without weighting procedures was used throughout.

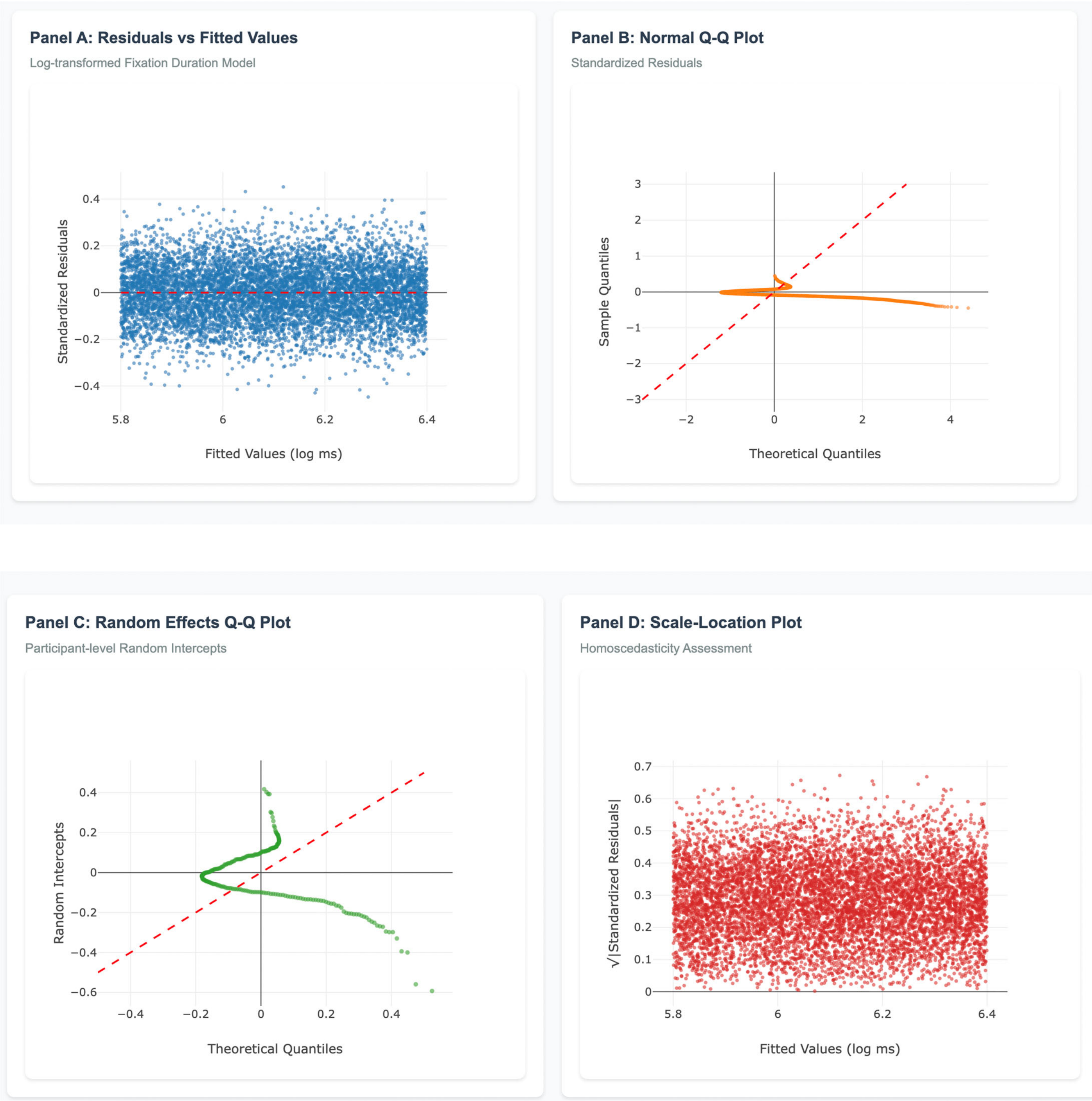

Figure S1. Model diagnostics.

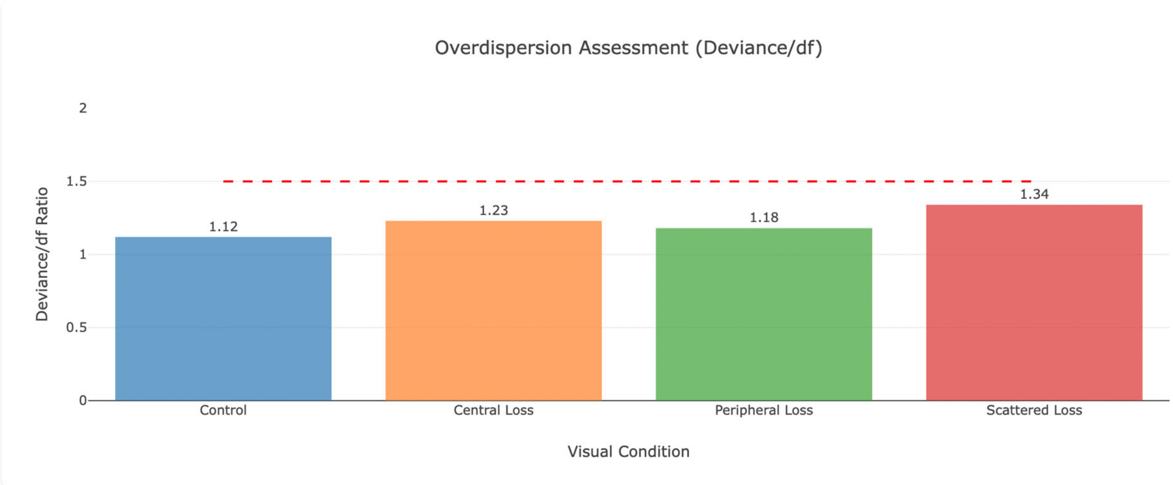

Figure S2. Overdispersion assessment.

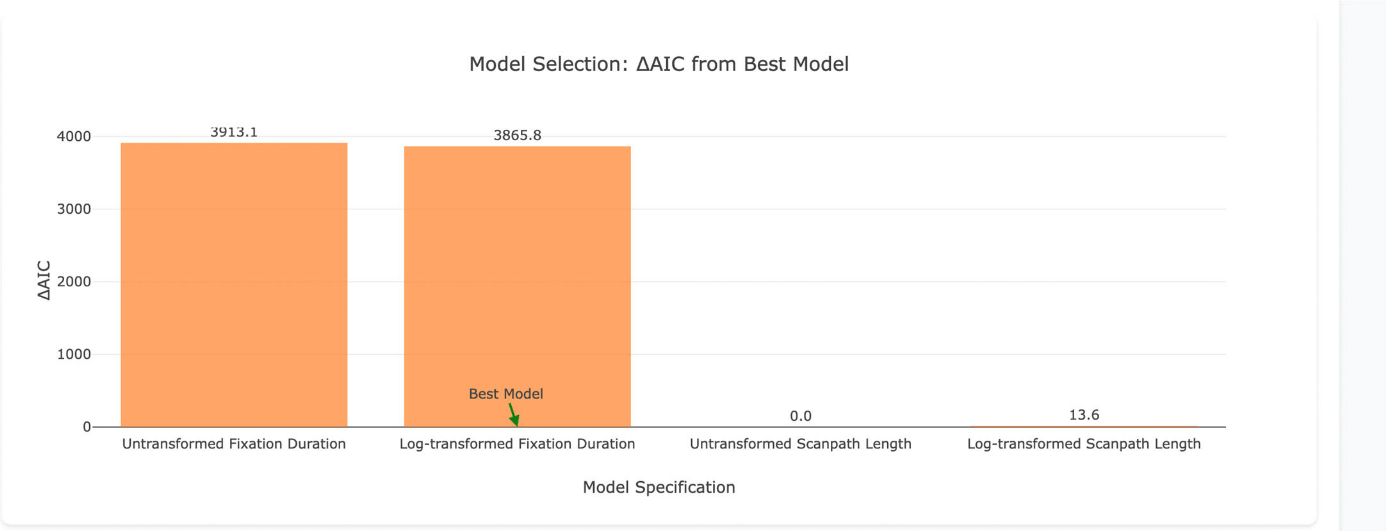

Figure S3. Model comparison.
